# Use of procalcitonin and white blood cells as combined predictors of infection in cardiac surgery patients

**DOI:** 10.1186/cc13405

**Published:** 2014-03-17

**Authors:** E Gomez, M Heredia, P Jorge, M Lorenzo, J Gomez-Herreras, E Tamayo, S Gutierrez, E Alvarez

**Affiliations:** 1 Hospital Clínico Universitario de Valladolid, Spain; 2 Hospital Río Hortega, Valladolid, Spain

## Introduction

The aim of this study is to identify a marker able to predict sepsis in cardiac surgery patients and to differentiate SIRS from infectious and non-infectious origin. The occurrence of sepsis after cardiac surgery increases the mortality risk. Sepsis may be mistaken for the cardiac surgery-associated systemic inflammatory response syndrome (SIRS) [1].

## Methods

A prospective, observational study was carried out to compare procalcitonin (PCT), C-reactive protein (CRP) and white blood cells (WBCs) during the first 10 postoperative days after cardiac surgery with cardiopulmonary bypass (CPB) between 122 patients with infection (Infection group) and 301 without (Control group) to identify predictors of infection.

## Results

The WBC and PCT median values were significantly (*P *< 0.05) higher in infected than control patients, during 10 postoperative days and on the third and fourth postoperative day, respectively, both variables showing a peak at 3 days in infected patients. The number of times that the WBC count surpassed its second postoperative median value (13,000 cells/mm^3^) during the first 3 postoperative days plus the number of times that PCT surpassed its own (1.7 ng/ ml) was a parameter (ranging from 1 to 6) with three categories (R1 = surpassed zero to one time; R2 = two to three times; R3 = four to six times) significantly associated with risk of infection, which increased with increasing number of times (R2: OR = 2.48 (1.32 to 4.65) and R3: OR = 7.06 (3.17 to 15.71)).

## Conclusion

Daily assessment of WBC and PCT during the first 3 postoperative days may be of value to diagnose and predict infection in cardiac surgery patients.
